# Ecological momentary assessments and interventions for youth substance use across clinical settings: A systematic review

**DOI:** 10.21203/rs.3.rs-10525385/v1

**Published:** 2026-07-30

**Authors:** Jillian Halladay, Veena Hira, Kathryn Gex, Samuel Meisel, Zachary Bryant, Jack Wilson, Emily Stockings, Tim Slade, André McDonald, James MacKillop

**Affiliations:** McMaster University; McMaster University; Medical University of South Carolina; Boston University; University of Sydney; University of Sydney; University of Sydney; University of Sydney; McMaster University; McMaster University

**Keywords:** alcohol, cannabis, nicotine, adolescent, emerging adult, momentary, ecological, ecological momentary assessment

## Abstract

**Purpose:**

Preventing youth substance use and related harms is a global priority. Ecological momentary assessment (EMA) and ecological momentary interventions (EMIs), which assess and address substance use in real-time, may improve mechanistic understanding, screening, intervention in clinical care. This review synthesized EMA/EMI studies examining substance use among youth in clinical settings.

**Methods:**

A systematic narrative review (PsychInfo, Medline Scopus) identified EMA/EMI studies assessing substance use in clinical samples of youth (mean/median age ≤ 25) across emergency, medical, mental health, and substance use treatment settings.

**Findings:**

Of 12093 titles/abstracts and 2157 full texts, 43 studies of 24 unique protocols (15 observational, 9 intervention) were included. Alcohol (75%), cannabis (67%), and nicotine/tobacco (33%) were the most frequently studied substances. EMA protocols typically lasted 14 days with 4 daily prompts; 3 incorporated passive (bio)sensing. Studies included 270 momentary antecedents and 17 momentary consequences of substance use, organized into six domains: substance use, affect/cognitions, environmental context, social context, movement factors, and EMA compliance. Negative affect, contextual factors (e.g., evenings, weekends, friends), and prior substance use showed the most consistent associations with use, although findings were heterogeneous. Passive sensing approaches demonstrated strong predictive performance. Thirteen evaluated EMIs integrating real-time monitoring with feedback, goal-setting, and/or coping support reduced substance use harms.

**Conclusions:**

EMA/EMI approaches show promise for understanding and addressing dynamic, context-dependents substance use processes among youth in clinical settings. While the evidence remains exploratory and methodologically heterogenous, findings suggest EMIs can deliver timely, individualized support. Future research should prioritize greater methodological transparency and rigor.

## 1. Background

Given links between early substance use and increased morbidity across the lifespan – resulting in substantial personal, health system, and societal costs – preventing youth substance use and related harms is a global health priority (Degenhardt et al., 2016). Recent national surveys from high income countries suggest that about 1 in 5 people meet criteria for a lifetime substance use disorder (SUD), with point prevalence of SUDs highest among youth and recent increases in the severity and complexity of SUDs (Slade et al., 2024, Ten Have et al., 2023, Halladay et al., 2025). Over half of people first use a substance before 20 years of age, and although most substance use disorders emerge in the mid-20s, symptoms begin nearly a decade earlier (Solmi et al., 2021, Blanco et al., 2018). As such, adolescence (ages 12–17) and emerging adulthood (ages 18–25) represent critical windows for early identification and effective youth-tailored indicated prevention or treatment (Stockings et al., 2016).

Despite long-standing clinical guidelines recommending routine assessment and intervention for youth substance use in both medical and mental health settings, this practice remains limited (National Institute on Drug Abuse, 2014, Burkstein, 2020, Weitzman et al., 2025). Routine, repeated assessments is a similarly underutilized core component of behavioral treatment for youth SUDs (Spencer et al., 2021, Hogue et al., 2025). Emerging technologies that enable real-time, context-sensitive assessments and in-the-moment interventions, such as ecological momentary assessments (EMA) and ecological momentary interventions (EMIs), offer promising avenues to transform how we understand and respond to youth substance use (Votaw and Witkiewitz, 2021, Dora et al., 2023, Carpenter et al., 2016). These methods have potential to capture the dynamic interplay of emotional, social, and environmental factors that drive use, providing new opportunities for tailored, personalized interventions. EMA and EMI also have the capacity to be implemented across a diverse range of clinical and community services through which youth may seek substance use treatment,

Youth with substance use concerns access care in emergency departments (EDs), primary care, mental health programs, and specialized substance use programs. ED often serves as the first point of contact for substance use care, with emergency providers playing a critical role in identifying and reducing substance-related harms (Hawk et al., 2019). Primary care providers are well positioned to deliver ongoing, accessible care, particularly during transitions from pediatric to adult systems when SUDs commonly emerge (Schraeder et al., 2021). Youth who seek specialized care for SUDs typically have co-occurring mental health difficulties, and many have prior engagement with mental health services (Garey et al., 2020, Halladay et al., 2021, Turner et al., 2004, Hawkins, 2009).

EMA techniques collect in-the-moment repeated prospective longitudinal data in real-world settings. For the purposes of this review, we adopt a broad conceptualization that includes EMA and closely related intensive longitudinal approaches, including daily diaries, passive sensing, experience sampling methods, momentary sampling, and ambulatory assessments (Carpenter et al., 2016). Although distinctions exist between these approaches, each involves repeated measurement in participants’ natural environments over short timeframes. The term EMA is used throughout this review as an umbrella term for these methodologies. Due to the in-the-moment or short retrospective recall periods (< 1 day), EMA provides more accurate insight into the context, antecedents (i.e., factors that occur before), and consequences (i.e., factors that occur after) of substance use behaviours than other designs (i.e. panel data with wide sampling frames) given the minimization of recall bias, enhanced ecological validity, and ability to capture dynamic momentary processes (Votaw and Witkiewitz, 2021, Dora et al., 2023). These approaches are increasingly being used for behavioural monitoring, digital phenotyping, and mobile health interventions (Carpenter et al., 2016). A review of 168 EMA studies in adult psychiatric clinical samples found EMA to offer potential clinical utility related to enhancing patient conceptualization, treatment planning, personalized monitoring and feedback systems, treatment tracking, and tailoring or adjusting interventions to individual needs (Mink et al., 2025). Tailored interventions can include EMA-based treatment recommendations or EMIs, otherwise known as just-in-time adaptive interventions. EMIs are typically used to target in-the-moment behaviour changes, though most current EMI evaluations are pilots (Hsu et al., 2025). To date, there remains limited insight related to the antecedents, behaviours, and consequences (ABCs) linked to real-time substance use among youth in clinical settings, and related efficacy of clinically embedded EMIs.

Youth are highly engaged with technology, positioning EMAs and EMIs as promising approaches to personalize treatment, reduce clinician burden, and extend support beyond traditional face-to-face clinical encounters for youth. This holds particular promise given the current mental health and addictions workforce and systems are not equipped to meet the needs of youth (Sterling et al., 2010, Ohene-Addo, 2026, Lu et al., 2023). Despite this developmental fit and clinical promise, EMA/EMI research and practice has predominantly focused on alcohol use among non-clinical adult samples (Dora et al., 2023, Tovmasyan et al., 2022, Votaw and Witkiewitz, 2021), leaving significant gaps in understanding real-time substance use among youth. Youth substance use may be driven by numerous interconnected factors including affect, social environment, availability of substance-free activities, and ease of access to substance use (Dora et al., 2023, Votaw and Witkiewitz, 2021). Cannabis-focused EMA studies are also limited despite cannabis being the leading cause of substance-related service use among youth (Hawke et al., 2018, Halladay et al., 2023), and clinical samples are rarely examined despite evidence of differential momentary patterns when compared to general population samples (Votaw and Witkiewitz, 2021, Votaw et al., 2022). Synthesizing existing EMA/EMI research in youth clinical samples is thus essential to inform novel, personalized, targeted, and accessible developmentally-informed prevention and treatment strategies that bridge the gap between those who need and have access to effective substance use interventions.

### 1.1 Objectives

This systematic review provides a comprehensive overview of EMA and EMI studies that explore substance use in clinical samples of youth, including a narrative synthesis of: (1) sample and clinical setting characteristics; (2) EMA protocols; (3) antecedents, behaviours, and consequences (ABCs) of substance use and their momentary relationships to use; (4) types and effectiveness of EMIs; and (5) gaps and areas for future clinical research. These are reported separated by clinical setting.

## 2. Methods

### 2.1 Search

The current review is a sub-review of a larger parent review that aimed to provide a comprehensive overview of EMA methods that have been used to measure substance across all settings and samples (CRD42023400418). A systematic search was conducted up to April 9, 2026 in PsychInfo, Medline, and Scopus combining terms for EMA approaches and substance use (full search available in Supplementary Materials 1 [SM1]). Hand searching reference lists and citing forward were also done to identify additional papers. For this review, two reviewers independently assessed eligibility based on if articles: (1) assessed substance use behaviour(s) using EMA (i.e., repeated or ongoing data collection from digital devices); (2) were conducted in clinical settings including medical, mental health, or substance use care in emergency, inpatient, or outpatient clinics; and (3) included youth with mean/median ages < = 25. A third reviewer resolved discrepancies.

### 2.2 Data extraction

Two reviewers independently extracted information to an Excel document on: study and sample demographics, study design and data collection methods (e.g., assessment schedule), substance use measures, non-substance use measures (e.g., affect, cognitions, compliance, environment, social context, movement), theoretical approach (e.g., mapping to ABC framework), analytical approaches, summary of findings, and quality assessment. Information extracted related to the EMA assessment schedule included the type and timing of when participants were prompted to complete assessments, including time-contingent (fixed times), signal-contingent (random times), and event-contingent (initiated by youth based on a particular event, usually substance use) prompts as well as any passive sensing (data collected by devices, such as geolocation or typing speed). Adherence to these schedules was also extracted, including percentage of assessments completed, response latency (time between the prompt being sent and competed), and response windows (researcher-defined time allowed for responses before the assessment expires). Discrepancies were resolved through discussion or a third reviewer.

### 2.3 Quality assessment

Study quality assessed using the EMA checklist by Liao et al. (2016) which includes rating following four domains as weak, moderate, or strong: (1) strength of rationale for EMA design; (2) *a priori* power analysis; (3) adherence; and (4) treatment of missingness.

### 2.4 Synthesis

Results were synthesized descriptively and presented separately by clinical setting. For observational studies, momentary antecedents and consequences were grouped into higher-level theoretical domains. Associations between antecedents, substance use, and consequences were summarized by direction and significance in the text and tables. Microassays illustrate the antecedent categories assessed across studies, with counts and distributions of significant associations reported alongside each figure. Harvest plots accounting for study quality and nesting within studies are provided in SM10. For intervention studies, the direction and significance of EMI effects were summarized in the tables and text.

## 3. Results

12093 titles/abstracts and 2157 full-texts were reviewed, including 43 articles in the final review. This included 24 studies with unique protocols, and 19 secondary analyses. Of unique protocols, 15 were observational and 9 were intervention studies with a total participant pool of 3715 youth. Most studies were conducted in the USA (88%). See SM2 for PRISMA Flow and Table 1 for protocol characteristics.

### 3.1 Characteristics of EMA/EMI protocols

#### 3.1.1 Sample characteristics

Clinical settings included 7 (29%) emergency department, 6 (25%) medical outpatient programs, 5 (21%) mental health programs, and 6 (25%) substance use programs (Table 1). The median sample size was 47, ranging from 8 to 1131. Half of the studies were adolescent (50%) and half emerging adult samples, with an overall average age of 19.3 (SD = 3.2).

#### 3.1.2 Substance use ABCs

The most commonly targeted substances, defined as substances of focus assessed with EMA or addressed with EMI, included alcohol (75%), cannabis (67%), general substance use (42%), and nicotine/tobacco (33%) (Table 1). See SM6-SM7 for visualizations.

Observational studies typically explored momentary antecedents and/or consequences of substance use and related behaviours (n = 26/27).[1] Overall, there were 270 momentary antecedents, with 164 (61%) from machine learning [ML] models. Antecedents were organized into 6 higher order domains: substance use (15 antecedents; mainly comprised of cannabis or alcohol frequency or indicators of problematic use), affect and cognitions (41 antecedents; mainly comprised of positive and negative affect or use motives), environmental context (78 antecedents, 47 from ML studies; mainly related to location and time), social context (39 antecedents, 22 from ML studies; mainly related to who they were with and social experiences), movement factors (95 antecedents, all from ML studies; mainly collected through passive accelerometry monitoring), and EMA compliance (2 antecedents). Additionally, there were 17 total momentary consequences: 16 related to affect and cognitions and 1 compliance related. See Figs. 2–3 and SM9-SM10 for visualizations of ABCs.

All intervention studies incorporated an EMA protocol followed by EMIs triggered by momentary responses and explored whether the EMI changed substance use.

#### 3.1.3 Assessment methods and protocols

The median target assessment period was 14 days with 4 assessments per day. Almost all protocols used time-contingency sampling (73.9%), nearly two thirds (65.2%) used signal contingent approaches, one third (30.4%) used event contingent approaches, and only 3 (13%) used passive monitoring. Adherence was moderate with an average response rate of 69% to the prompts in the study (i.e., mean ~ 2.8/4 daily prompts completed). Observational protocols showed a wide range of adherence from 30–90% (mean = 64%). Intervention protocols commonly reported declining response rates over time with the average start response rate being 84% and the end being 69% (k = 5 reported start/end). Linear regressions were conducted to predict the average adherence (0–100%) for unique protocols (See SM4). Protocols that included both time- and signal-contingent sampling had significantly lower adherence (mean = ~ 59%) compared to those that did time-contingent only (~ 76%; p = 0.03). No other associations were statistically significant at p < 0.05.

Time to completion data were rarely reported, with 87% (k = 20) of protocols providing no information on response timing with only 20% of observational and no intervention studies reporting these data. Among the few reporting, latency varied widely including 2.5 mins, 8 minutes to 3 hours, and 30.5% completing on the same day as the prompt. Studies that provided response windows for time-contingent prompts ranged from 8 minutes to 3.5 days with a median of 40 minutes (k = 8). Studies that provided response windows for signal-contingent prompts ranged from 8 minutes to 1 day, with a median of 15 minutes (k = 7).

#### 3.1.4 Study quality

**Figure 1:**
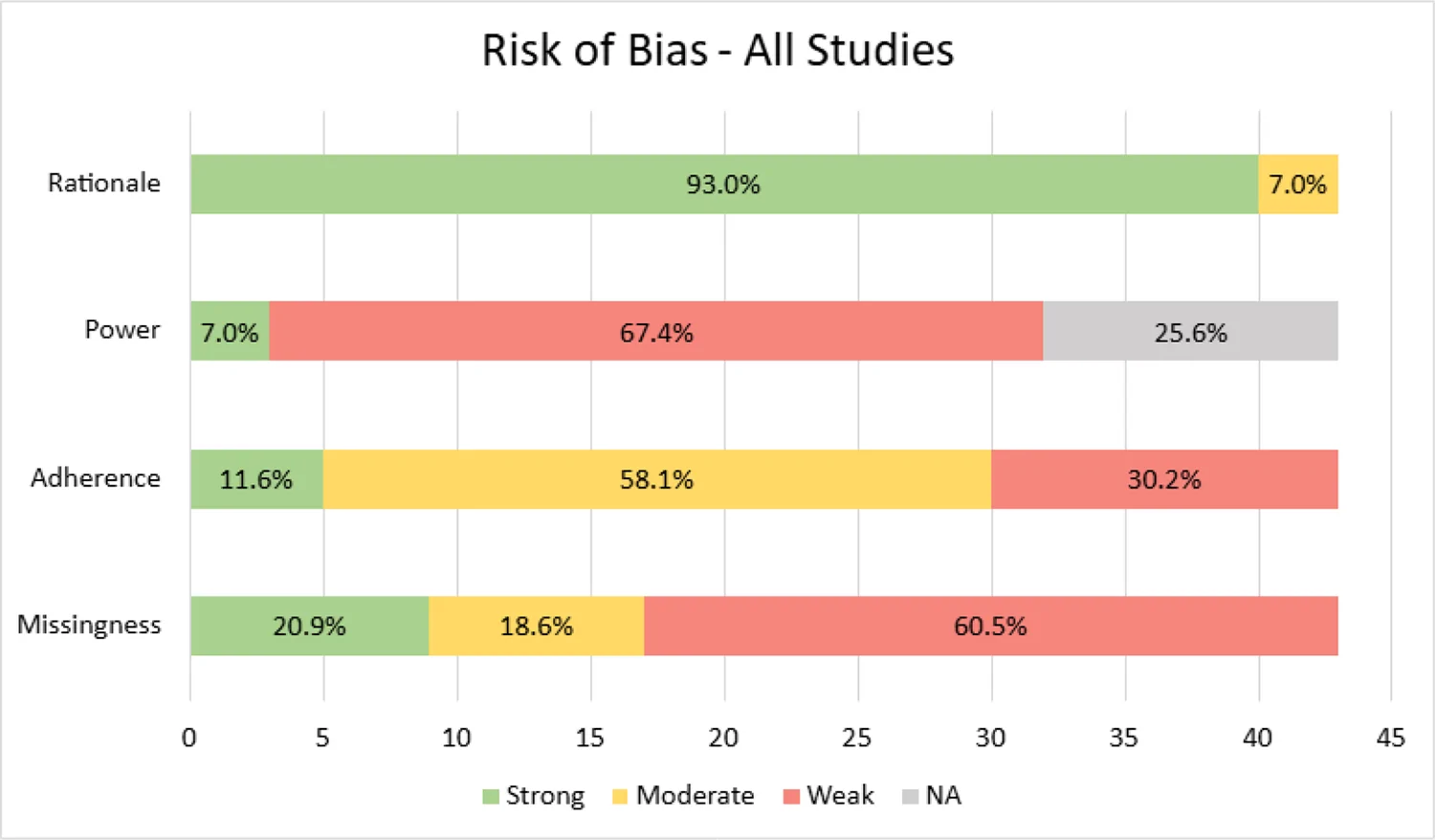
Distribution of risk of bias assessments across all studies k=43).

**Figure 2:**
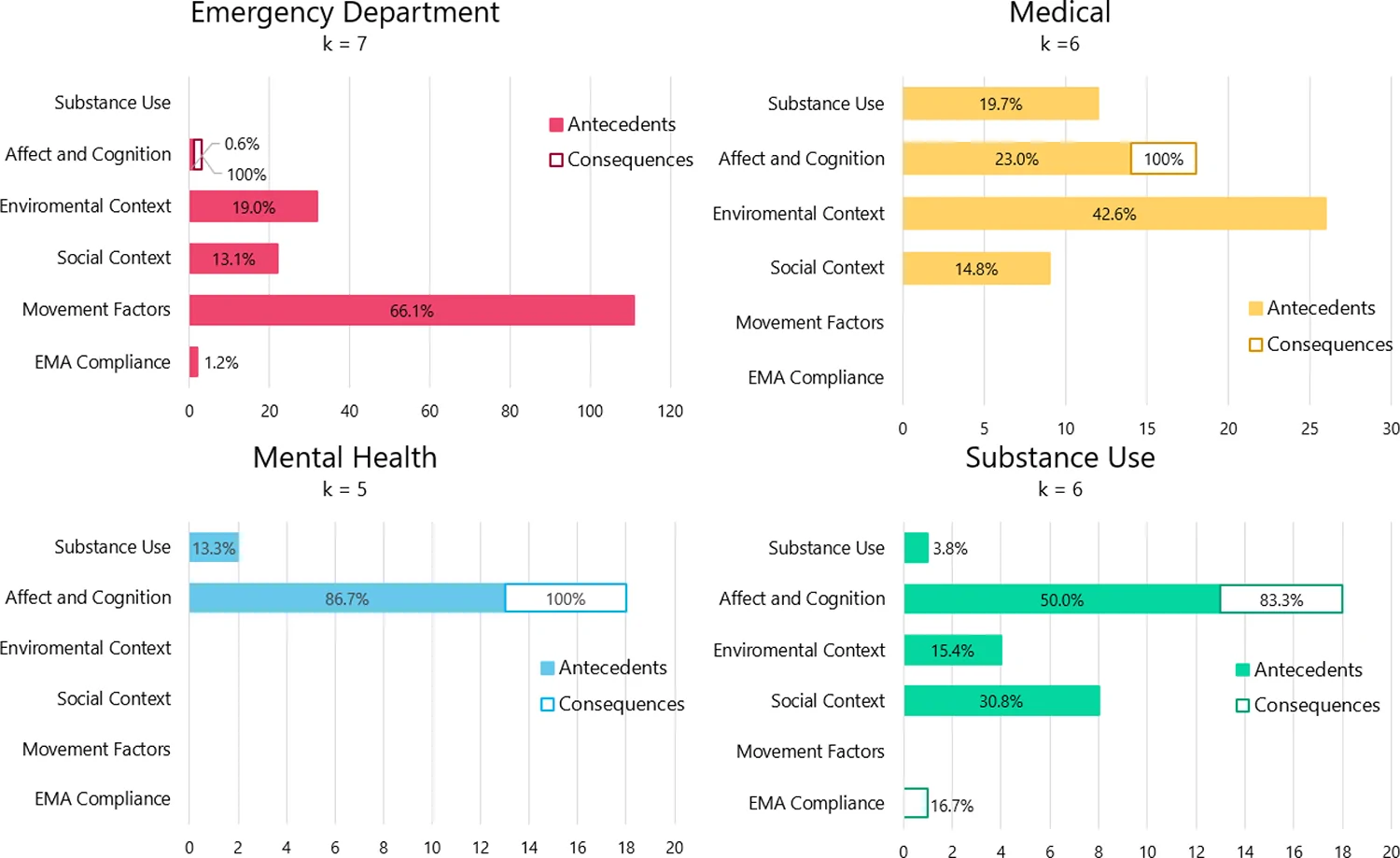
Distribution of momentary antecedents and consequences of momentary substance-related behaviours. This stacked bar graph displays the number of variables (n, horizontal axis) categorized by type of momentary antecedents and consequence across observational studies with a substance use behaviour as the target outcome (n=26). Percentages on bars were calculated out of the number of antecedents (totals to 100%) and consequences (total to 100%) within clinical settings.

Across all studies, most provided strong justification for use of EMA (93%). Few conducted power calculations (< 10%), though one quarter (26%) were pilot/feasibility studies where power calculations are not applicable. Protocol adherence varied (as reported above), with only 11% reporting strong adherence (i.e., > 80%). Missing data was accounted for in 21% of studies with 30.2% not reporting handling of missing data. See Fig. 1 for risk of bias across all studies and SM5 separated by study sub-types.

### 3.2 Findings across clinical settings

**Figure 3:**
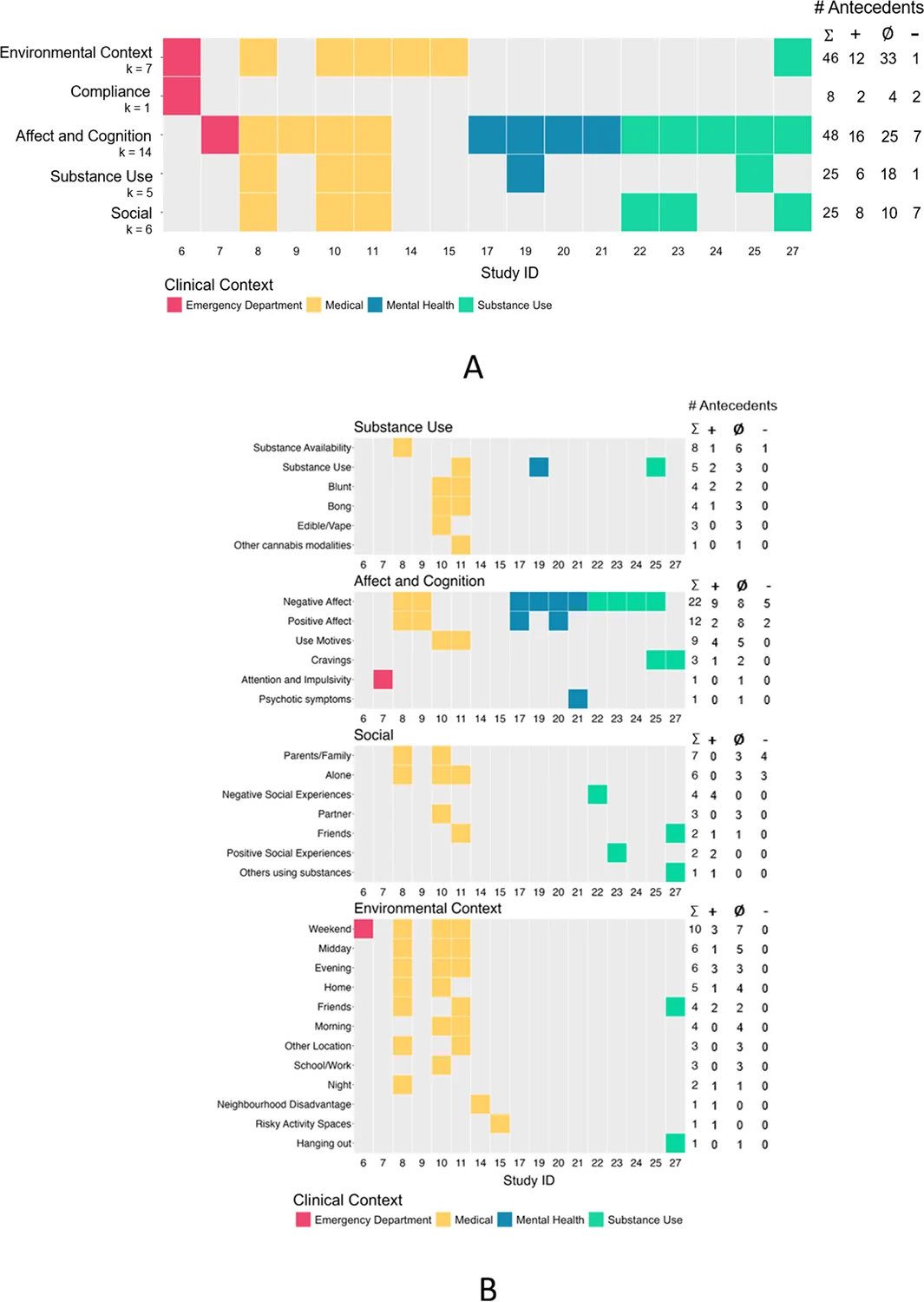
**A:**Microassay of antecedents in observational studies by higher order domain (n = 152 antecedents) Microassay of higher order domains of antecedents, where y-axis shows domains of antecedents and k number of studies in each domain, x-axis shows study ID, and secondary y-axis shows total number of antecedents per domain, and number of antecedents with increasing (+), null (∅), or decreasing (−) associations with substance use-related behaviours. Antecedents with descriptive or non-reported associations were not included. **B:** Microassay of observational studies split by lower order domains (n = 152 antecedents reporting statistical results, not including ML studies) Microassay split by lower order domains, where y-axis shows name of specific antecedent, x-axis shows the Study ID and secondary y -axis displays the total number of antecedents (∑), and number of antecedents with increased (+), null (∅) and decreased (−) associations with substance-related behaviours. Antecedents with descriptively or non-reported associations were not included.

Descriptions of observational studies are presented in Table 2 and intervention studies in Table 3. Figure 3 summarizes outcomes for observational momentary antecedents. Where formal statistical testing was conducted on associations between momentary antecedents and substance use, the proportion of significant associations was highest for social context (60%), followed by environmental context (50%), and affect and cognition (48%), with lower proportions observed for substance use-related antecedents (28%). When looking at specific variables, negative affect (n = 22 antecedents), positive affect (n = 12), and day of the week (n = 10) were most commonly explored (Fig. 3A). Substance use was generally more common on weekends and evenings, and less common with parents/family or alone than with friends/others. Findings for negative affect were mixed, with nine antecedents showing increases (41%), eight no association, and five decreases in substance-related outcomes. Positive affect was less commonly explored, though yielded a higher percentage of null associations (8/12, 67%) than negative affect (8/22, 36%; Fig. 3B). These findings varied by clinical setting, detailed below.

#### 3.2.1 Findings within emergency department (ED) settings

EDs often serve as brief, single-episode points of contact focused on acute stabilization. Across studies, youth were identified in ED through substance use screening, with most actively excluding youth presenting for substance-specific concerns.

##### 3.2.1.1 Observational studies

Seven observational studies recruited youth from EDs, primarily focused on alcohol use with one on cannabis. Sample sizes ranged from 10 to 296. Four studies used ML models applied to large volumes of passively collected mobile-device data, including environmental (time, day, geolocation), social factors (texting/calling patterns), and movement factors (geolocation, accelerometry, keypad interactions) (Bae et al., 2018, Bae et al., 2017, Bae et al., 2023, Suffoletto et al., 2018b). These passive sensing approaches demonstrated high accuracy in predicting heavy drinking episodes, with performance ranging from 84–97%. The remaining three studies examined cognitive, environmental, and methodological factors. Momentary attention and approach biases did not predict heavy episodic drinking (Suffoletto et al., 2020) while higher blood alcohol concentration reduced information processing without affecting visuo-spatial working memory (Suffoletto et al., 2017). Measurement-focused work showed that substance use reporting varied by assessment timing and methods: participants drank more on weekends than weekdays, while cannabis use remained consistent; daily assessments prompted higher reports of alcohol use overall but lower quantities than weekly assessments; and interactive voice response yielded fewer cannabis use episodes but higher reported quantities compared to SMS (Buu et al., 2020).

##### 3.2.1.2 Intervention studies

Three intervention protocols tested EMIs for alcohol use within ED-recruited samples, where sample sizes ranged from 38 to 938. The Texting to Reduce Alcohol Consumption (TRAC) trial showed that asking youth about weekend drinking plans and providing real-time feedback encouraging low-risk drinking plans reduced binge drinking compared to assessment alone or no assessments (Suffoletto et al., 2014, Suffoletto and Chung, 2016). The TRAC2 trial extended this by using an algorithm to set personalized drinking limit goals, finding reductions in drinking consequences strongest for youth who engaged longer, with participants preferring goals that gradually reduced their alcohol use compared to abrupt/large suggestions for change (Suffoletto et al., 2018a).

The MATCH trial provided the most rigorous comparison of an EMI, testing four different active EMA/EMI apps – focusing on PLANs to drink, personalized feedback related to USE, GOAL-setting exercises and feedback, or a combination of these EMIs (COMBO) against a self-monitoring control (TRACK). Youth in the USE, GOAL, and COMBO arms (but not PLAN) showed significant reductions in alcohol use and/or consequences compared to TRACK control (Suffoletto and Chung, 2025, Suffoletto and Chung, 2023a). There was some evidence these changes were mediated by decreases in desire to get drunk, and desire to get drunk was associated with greater odds of drinking, heavy drinking, and lower goal commitment and confidence (Suffoletto and Chung, 2023a, Suffoletto and Chung, 2023b). Another included article from the MATCH trial identified pre-intervention characteristics (including sex, race, perceived risk of binge drinking, perceived peers intoxication, and cannabis use) that may help match youth to more effective, personalized EMIs within MATCH (Chung et al., 2025).

##### 3.2.1.3 ED summary

ED-based research shows strong promise for digital prediction and intervention using EMA/EMI: passive sensing can accurately detect high-risk drinking, while brief, tailored EMIs meaningfully reduce alcohol-related harms. Observational work further found self-reported cognitive factors offered little predictive value, and self-reports vary by assessment timing and mode. However, little is known about other antecedents and consequences related to affect, motives, and other substance use and there are no EMA/EMI studies related to higher-risk youth who present to the ED for acute substance-related concerns.

#### 3.2.2 Findings within medical outpatient settings

Medical outpatient settings (largely primary care) provide ongoing health care and serve as key settings for the early identification and treatment of emerging mental health and substance use concerns. Across studies in medical outpatient settings, youth were identified through substance use screening rather than substance-specific presentations.

##### 3.2.2.1 Observational studies

Nine observational studies recruited youth from outpatient medical settings, with 6 focused on cannabis use, two general substance use, and one on alcohol. Sample sizes ranged from 8 to 248.

For cannabis-related studies, EMA studies demonstrated feasibility and acceptability (Black et al., 2014) and explored affect and cognition, environmental, social, and substance-related factors. Cannabis use was associated with prior and subsequent negative, but not positive, affect (Shrier et al., 2014, Ross et al., 2018). However, shifts in affect after cannabis use were found to be dependent on whether an individual experienced cannabis dependence, with more positive affect post-use for those with dependence, and more negative affect for those without (Ross et al., 2018). Motives were generally unrelated to momentary use (Shrier et al., 2013), though using to conform was associated with greater affective shifts (Ross et al., 2018) and motives frequently changed from pre- to post-use, particularly when external (i.e., social and conformity motives; (Shrier and Scherer, 2014).

For cannabis, contextual factors showed modest effects: evenings and weekends were linked to greater dose and craving, but not intensity or duration of high (Shrier et al., 2013, Shrier et al., 2012). Social context was mostly null, with lower-intensity use and weaker craving when alone or with parents (vs. friends). Affect was not consistently related to craving occurrence, but both positive and negative affect were associated with greater craving strength, which was also higher in less structured settings (e.g., home, friends’ houses) (Shrier et al., 2012).

For alcohol, negative affect, using to get drunk or cope, and being with a partner were associated with use (Kauer et al., 2009). For any substance use, broader environmental factors were explored, including neighbourhood disadvantage and risky activity spaces linked with more use (Mennis et al., 2016, Mason et al., 2016).

##### 3.2.2.2 Intervention studies

The MOMENT intervention was the only EMI implemented in a medical outpatient setting. The trial included 248 youth to evaluate an in-person motivational enhancement therapy (MET) session alone compared to MET followed by two weeks of self-monitoring with (MOMENT) and without (EMA-only) supportive messages related to individuals’ personal triggers for cannabis (Shrier et al., 2018). Cannabis use was lower at 3-month follow-up time for those receiving MOMENT (MET + EMI) compared to MET-only, and cannabis craving were lower for those receiving MOMENT compared to MET-only (Shrier et al., 2018). MOMENT increased mindfulness attention, which was not found among those receiving MET-only, with mindfulness attention being related to lower negative affect and cannabis craving (Shrier and Harris, 2023).

##### 3.2.2.3 Medical outpatient summary

Outpatient studies showed selective, outcome-specific associations. Cannabis use was most consistently linked to negative affect, while motives and social context were largely unrelated; however, motives shifted across use episodes. Contextual factors (e.g., evenings, weekends, less structured settings) were modestly associated with greater use and craving intensity. Evidence beyond cannabis was limited but similarly pointed to negative affect and broader environmental risk. One EMI, focused on supportive messages when experiencing triggers for use, showed reduced cannabis use and craving when added to MET.

#### 3.2.3 Findings within mental health clinical settings

Mental health settings provide inpatient and outpatient services for a range of psychiatric disorders, often not specializing in SUD treatment. Accordingly, included studies sampled youth with non-SUD psychiatric disorders. These settings offer important opportunities to identify emerging substance use problems, given the high co-occurrence of mental health and substance use concerns and the psychotherapeutic expertise of clinicians in these contexts.

##### 3.2.3.1 Observational studies

There were five observational studies conducted in mental health settings, including outpatient and following inpatient or partial hospitalizations. Sample sizes ranged from 19 to 62. Most studies explored alcohol and/or cannabis use among youth experiencing internalizing-related symptoms or disorders including depression, anxiety, and suicidality (Brick et al., 2023, Bhushan et al., 2013, Selby et al., 2014, Stevenson et al., 2022). One sampled youth at high risk for psychosis and assessed substance use more broadly (Weiss et al., 2022). All studies focused exclusively on substance-related and affect- or cognition-related momentary antecedents or consequences. Concurrent drug use was found to predict cannabis use (Brick et al. (2023), and alcohol (but not other drug use) predicted self-injury (Selby et al., 2014). Most affect-related antecedents showed null or inverse relationships with substance use (Bhushan et al., 2013, Stevenson et al., 2022, Weiss et al., 2022), though substance use was found to be positively associated with later self-injury (Selby et al., 2014); alcohol) and negative affect (Weiss et al., 2022); general substance use). Notably, Weiss et al. (2022) did not find psychosis symptoms to be a significant antecedent or consequence of general substance use. Further, while Stevenson et al. (2022) largely found null relationships between affect and substance use in the full sample, and many did not experience affect-driven pathways to substance use (58%), there were subsamples of youth who experienced positive− (21%) and negative− (16%) affect driven pathways to alcohol use.

##### 3.2.3.2 Intervention studies

One single-arm EMI targeted alcohol use among youth post-discharge from mental health partial hospitalization called Project CHOICE (Choosing Healthy Options in Coping with Emotions) (Blevins et al., 2021). Project CHOICE incorporated EMA and EMI whereby those reporting negative mood or intention to drink received an individualized coping message based on an initial in-person session with a clinical psychologist. Twenty youth engaged with this EMA/EMI after discharge for 6 weeks, showing significant reductions from baseline in alcohol use, problems, and drinking to cope. There was generally a weaker association between negative mood and alcohol use at the end of the intervention period, and those who used more coping strategies were less likely to drink in response to negative mood (Blevins et al., 2021, Stevenson et al., 2020).

##### 3.2.3.3 Mental health settings summary

Evidence from mental health clinical settings suggests limited and heterogeneous momentary associations between affect and substance use. Concurrent substance use predicted use and use increased risk for adverse mental health-related outcomes such as self-injury. Most affect-related antecedents showed null or inverse associations with use overall, though smaller subgroups showed significant positive- or negative-affect-driven pathways. Intervention evidence is limited but promising, with one EMI reducing alcohol use and coping-motivated drinking post-discharge. Current evidence is focused on internalizing and psychotic symptoms, with no exploration of externalizing concerns and limited integration of environmental or social factors.

#### 3.2.4 Findings within substance use treatment settings

Substance use settings provide specialized assessment and treatment for substance-related concerns. Across studies, youth were recruited directly from treatment programs based on current or recent substance use, often as part of routine care within the program or during discharge or transition planning.

##### 3.2.4.1 Observational studies

Six observational studies examined youth attending substance use programs, where sample sizes ranged from 28 to 55. Four focused on emerging adults in collegiate recovery centres (primarily assessing craving) and two focused on adolescents in outpatient treatment (assessing any substance use). Among emerging adults, craving was generally preceded by negative affect, prior day smoking, and both positive and negative social experiences (Cleveland and Harris, 2010, Zheng et al., 2013, Zheng et al., 2015, Suffoletto and Chung, 2025). Craving was also associated with subsequent increases in negative affect and craving (Zheng et al., 2013, Zheng et al., 2015). The association between negative affect and later craving was amplified by avoidant coping and attenuated by problem-solving coping (Cleveland and Harris, 2010). Among adolescents in outpatient treatment, substance use was descriptively more likely during evenings and weekends, in the presence of friends or others who use substances, and at a friends’ house (Comulada et al., 2016). Daily craving was associated with greater substance use, where momentary affect showed inconsistent relationships. Substance use motives also appeared to vary by substance, with wanting to “get buzzed” associated with greater alcohol use and wanting to “relax” with greater cannabis use (Comulada et al., 2016). Additionally, momentary substance use among adolescents was related to lower EMA compliance (Comulada et al., 2015).

##### 3.2.4.2 Intervention studies

Four EMIs recruited youth from substance use programs, three in outpatient settings and one post-discharge for residential treatment. Sample sizes ranged from 29 to 200. One trial found using a mobile diary application for self-monitoring of cannabis use, which is common homework in substance use treatment, resulted in higher completion rates than a paper-based self-monitoring diary (Pitrat et al., 2024). The SMOKING trial, conducted in an outpatient setting, found that a text-based motivational interviewing and social network counselling intervention reduced the associations between baseline nicotine dependence and experiences of momentary stress on craving compared to those receiving general health-based text messages (Mason et al., 2015a, Mason et al., 2015b). An ecological momentary motivational enhancement therapy (EM-MET) intervention in an outpatient setting, combining four MET sessions with 2 weeks of EMA monitoring of person-specific triggers for use that prompted in-the-moment phone calls from therapists, significantly reduced cannabis temptation and dependence severity compared to METs alone (Darharaj et al., 2025). Lastly, a single-arm trial evaluated the Addiction Comprehensive Health Entrance Support System (ACHESS) for youth after residential treatment (Dennis et al., 2015). ACHESS prompted reflection on recent (past 30 minutes) emotions, activities, context, craving, and substance exposure, with on-demand EMIs focused on coping strategies. Using two or more EMIs within an hour of an EMA was associated with lower substance use in the following 7 days (Dennis et al., 2015).

##### 3.2.4.3 Substance use treatment setting summary

Among youth in substance use programs, momentary craving, affect, and social context appear to be important correlates, and possible intervention targets, of substance use, with patterns varying by developmental stage and setting. Among emerging adults, craving was consistently linked to negative affect, prior use, and social experiences, with evidence of bidirectional relationships and moderation by coping strategies. In contrast, adolescent findings emphasized contextual and social influences, with less consistent associations with momentary affect and stronger roles for daily craving and substance-specific motives. Intervention studies provide preliminary support for EMA/EMI approaches, particularly those integrating real-time monitoring, tailored feedback, and coping-focused support, which may reduce craving-related processes and substance use.

## 4. Discussion

### 4.1 Main findings

This review synthesized emerging evidence on the use of ecological momentary assessment (EMA) and ecological momentary interventions (EMIs) seeking to understand and address youth substance use across clinical settings. Observational findings highlight substantial heterogeneity in momentary predictors of substance use, with associations varying by domain, substance, and clinical context. While negative affect, prior substance use, social context, and temporal factors (e.g., evenings and weekends) were the most frequently identified correlates of momentary substance use, no antecedent consistently predicted substance use across all studies. Intervention studies showed relatively consistent evidence that EMIs integrating real-time monitoring with tailored feedback, goal-setting, coping support, and motivational approaches have potential to reduce substance use and related harms. Notably, studies varied considerably in their assessment schedules, constructs measured, adherence rates, and reporting practices, highlighting both the flexibility of EMA methods and challenges synthesizing findings. Overall, EMA/EMIs show promise for capturing and intervening on dynamic substance use processes among youth accessing care, although the evidence base remains preliminary and exploratory.

EMA and EMI findings related to affect and coping processes appeared to diverge. In the current review, negative affect was more consistently associated with increased substance use than positive affect, though findings were mixed with many null and occasionally inverse associations. These findings are relatively consistent with meta-analyses and systematic reviews of EMA studies that indicate no association between negative affect and use and only an association between positive affect and substance use in non-clinical, largely adult, samples (Dora et al., 2023, Tovmasyan et al., 2022, Votaw et al., 2022, Wycoff et al., 2018). In contrast, EMIs targeting affective processes, such as Project CHOICE (Blevins et al., 2021, Stevenson et al., 2020) and ACHESS (Dennis et al., 2015), demonstrated that responding to negative affect and promoting adaptive coping in the moment can reduce substance use. This discrepancy across EMA and EMI studies may reflect the field’s narrow focus on a simplified negative reinforcement model that assumes a direct, consistent, and universal link between negative affect and substance use (Carpenter et al., 2026). Together, these findings suggest that affective dysregulation remains an important intervention target, but that clarifying affective-substance use associations will require EMA studies to take a deeper, more nuanced, individualized approach that accounts for interactions with contextual, alternative reinforcers, and examines the effects of use on affect across individuals, situations, and clinical settings (Carpenter et al., 2026).

This review extends beyond affect to highlight the importance of environmental and social context. Across settings, substance use was more likely on weekends and evenings, in the home or at friends’ houses, and when with friends or partners vs. alone or with family. Emerging passive sensing studies further demonstrated strong predictive performance of heavy alcohol use using environmental (e.g., time, day, number of locations), social (e.g., calls/texts), and device movement (e.g., speed/rotation, screen unlocks, text deletions/insertions) data. EMIs directly targeted these social and environmental contexts. The TRAC/TRAC2 and MATCH trials intervened on these high-risk contexts (e.g., weekend drinking) by offering planning and feedback support to promote lower-risk substance use (Suffoletto et al., 2014, Suffoletto and Chung, 2016, Suffoletto et al., 2018a, Suffoletto and Chung, 2025, Suffoletto and Chung, 2023a, Chung et al., 2025). The SMOKING trial included a social network counselling component (Mason et al., 2015a, Mason et al., 2015b) and the MOMENT, EM-MET, and ACHESS interventions supported youth in identifying and responding to environmental and social contextual triggers (Shrier et al., 2018, Shrier and Harris, 2023, Darharaj et al., 2025, Dennis et al., 2015). These findings show the value in assessing and addressing the environmental and social context of youth substance use.

### 4.2 Clinical implications

These findings have important implications for clinical care. First, the observed heterogeneity in momentary associations supports conceptualizing youth substance use as a set of individualized, context-sensitive processes rather than uniform pathways (Hines et al., 2015, Trucco, 2020). EMA therefore has strong potential to enhance measurement-based care systems, which use structured, repeated assessments to guide treatment decisions, improve engagement, and monitor outcomes (Hickie et al., 2019, Lewis et al., 2019, Peterson et al., 2018, Scott and Lewis, 2015). Second, EMIs may offer a scalable way of extending care beyond face-to-face clinical encounters, by serving as treatment adjuncts in settings where clinicians do not have the skills, confidence, or time to deliver substance use interventions. Despite variability in observational findings, EMIs using motivational approaches, with or without the self-identification of triggers, focused on substance-free coping, planning, and/or goal setting demonstrated relatively consistent clinical benefits across settings. These findings suggest that EMIs do not require perfectly consistent momentary predictors to be effective, but rather can capitalize on timely, individualized intervention delivery. Embedding EMA/EMIs within care could support more personalized, precision medicine approaches that leverage youth engagement with technology and reduce clinician burden.

### 4.3 Limitations and future directions

Several methodological limitations should be considered. Studies varied widely in design, sample characteristics, construct coverage, substances targeted, and analytic approaches, limiting direct comparability across settings. Many studies were pilot or feasibility trials with relatively small samples, few reported power calculations, reporting on compliance and handling missing data were inconsistent, response timing and latency varied widely, and overall adherence was moderate, declining over time. Aligned with findings from this review, rigorous adherence to EMA protocols and generally poor reporting practices are known concerns in the field (Jones et al., 2019, Wrzus and Neubauer, 2023, Drexl et al., 2025). Future research should prioritize transparently report methods and protocol adherence using established reporting guidelines and templates (Kirtley et al., 2021, Liao et al., 2016, Trull and Ebner-Priemer, 2020, van Roekel et al., 2019, Hsu et al., 2025). With these considerations in mind, current findings should be interpreted as exploratory and used to inform further theoretical or clinical-based EMA/EMI research.

#### 4.3.1 Are the right constructs being measured?

While the constructs assessed across studies appear clinically relevant, the substantial heterogeneity of findings across many antecedents raises a broader question: are we measuring the processes most relevant to understanding momentary youth substance use? For example, although affect was commonly assessed, understanding how youth respond to, regulate, and cope with emotional experiences may be more informative than affective states alone. EMI findings demonstrating effective engagement with coping strategies supports this idea. Similarly, while studies frequently assessed social context and activity engagement, they rarely examined whether youth felt connected to others, experienced a sense of belonging, or found their activities meaningful, enjoyable, or rewarding. These distinctions may be particularly important given that substance use occurs within a broader system of competing reinforcers, coping strategies, and social experiences that are likely more relevant to clinical care than simply the presence of specific cues (Carpenter et al., 2026, Halladay et al., 2024, Trucco, 2020). Future research should move beyond identifying simple predictors for substance use and instead seek to understand how youth experience, interpret, and respond to these cues in everyday life.

#### 4.3.2 Tailoring protocols and expectations to the clinical setting

Across studies in the current review, adherence was moderate, declined over time, and tended to be lower for protocols using both time- and signal-contingent reports compared to time only. This highlights a potential trade-off between data resolution, participant burden, and engagement. However, identifying design features that reliably improve adherence remains challenging (Jones et al., 2019, Wrzus and Neubauer, 2023, Drexl et al., 2025). Emerging passive monitoring approaches may help address some gaps in self-report data while reducing participant burden. As the field develops, guidance for matching EMA designs to specific research and clinical objectives (e.g., predicting imminent substance use, monitoring skill use, characterizing recovery) may be more valuable than single standardized protocols aimed at maximizing adherence. More fundamentally, the field may need to reconsider whether adherence should be viewed as a universal marker of EMA/EMI quality. Moderate adherence should be expected in youth clinical populations given developmental stage, competing demands on attention and time, symptom burden, potential shift in access to devices (e.g., school, jail, hospitalizations), and the complexity of the clinical challenges many young people face. In contrast, lower engagement in EMIs may not necessarily indicate poorer outcomes. Some youth may disengage because they have learned the skills being taught, achieved their goals, or no longer perceive additional monitoring or intervention as beneficial.

## 5. Conclusion

EMA and EMI approaches offer promising tools for understanding and intervening on real-world substance use processes among youth accessing care. While observational findings revealed heterogeneity in antecedents and consequences, intervention studies demonstrated meaningful potential for reducing substance use through timely, personalized support. Advancing this field will require greater methodological consistency and transparency, stronger integration of contextual and experiential factors, and evaluation of implementation within routine clinical care.

## Supplementary Material

Supplementary Files

This is a list of supplementary files associated with this preprint. Click to download.

- HalladayEMAReviewSuppMats28July2026preprint.docx

## Figures and Tables

**Table 1:** Study and sample summary statistics of parent studies

Study Characteristics	Overall (n = 24)	Observational (n = 15)	Intervention (n = 9)
**Sample characteristics**			
**Country n (%)**			
USA	21 (87.5%)	14 (93.3%)	7 (77.8%)
Australia	1 (4.2%)	1 (6.7%)	0 (0%)
France	1 (4.2%)	0 (0%)	1 (11.1%)
Iran	1 (4.2%)	0 (0%)	1 (11.1%)
**Clinical Sample n (%)**			
Emergency Department	7 (29.2%)	4 (26.7%)	3 (33.3%)
Medical Outpatient	6 (25%)	5 (33.3%)	1 (11.1%)
Mental Health Program	5 (20.8%)	4 (26.7%)	1 (11.1%)
Substance Use Program	6 (25%)	2 (13.3%)	4 (44.4%)
**Sample size at baseline**			
**Total sample (N)**	3715	1198	2517
Mean (SD)	159.79 (265.46)	79.87 (95.55)	279.67 (398.2)
Median [min-max]	47 [8–1131]	44 [8–307]	50 [20–1131]
**Age**			
Mean (SD)	19.27 (3.2)	19.4 (3.1)	19.0 (3.4)
Median [min-max]	19.14 [12–29]	18.7 [12–29]	21 [13–25]
Adolescent sample n(%)	12 (50%)	8 (53.3%)	4 (44%)
Emerging adult sample n(%)	12 (50%)	7 (46.7%)	5 (56%)
**% Female** Median [min-max]	56.5 [12.9–86.7]	60 [29–86.7]	55 [12.9–70]
**% Race**^1^ Median [min-max]			
White	26.5 [0–98]	27 [0–98]	21 [0–85]
Black	28.3 [0–91]	15 [0–89]	43 [0–91]
East/Southeast Asian	0 [0–5.3]	0 [0–1.7]	0 [0–5.3]
Hispanic	0 [0–93]	0 [0–93]	0 [0–25]
Multiracial	0 [0–24]	0 [0–11.7]	0 [0–24]
Other*	0 [0–87.1]	0 [0–87.1]	2.8 [0–75]
**Substance(s) of interest**^4^ **n(%)**			
Alcohol	18 (75%)	12 (80%)	6 (66.7%)
Cannabis	16 (66.7%)	10 (66.7%)	6 (66.7%)
General substance use	10 (41.7%)	7 (46.7%)	3 (33.3%)
Nicotine/Tobacco (including cigarettes)	8 (33.3%)	4 (26.7%)	4 (44.4%)
Opioids	4 (16.7%)	3 (20%)	1 (11.1%)
Stimulants (cocaine/prescription stimulants, MDMA, meth)	3 (12.5%)	3 (20%)	0 (0%)
Psychedelics (LSD/acid)	2 (8.3%)	2 (13.3%)	0 (0%)
Sedatives	1 (4.2%)	1 (6.7%)	0 (0%)
**EMA Characteristics**			
**Study duration (Target assessment period in days)** ^2^			
Mean (SD)	20.6 (16.9)	16.3 (8.0)	27.9 (24.8)
Median [min-max]	14 [4–84]	14 [7–30]	15 [4–84]
**Number of assessments per data collection day**			
Mean (SD)	3.5 (2.2)	3.0 (1.7)	3.0 (2.1)
Median [min-max]	4 [1–6]	4 [1–6]	3.0 [1–6]
**EMA sampling design n(%)**			
Burst design	8 (33.3%)	4 (26.7%)	4 (44.4%)
Daily consecutive sampling	14 (58.3%)	10 (66.7%)	4 (44.4%)
Combined	2 (8.3%)	1 (6.7%)	1 (11.1%)
**EMA delivery n(%)**			
Text-based	9 (37.5%)	4 (26.7%)	5 (55.6%)
App-based	14 (58.3%) 1 (4.2%) used both app and text	10 (66.7%) 1 (6.7%) used both app and text	4 (44.4%)
Personal phone	12 (50%)	9 (60%)	3 (33.3%)
Study phone	12 (50%)	6 (40%)	6 (66.7%)
**Training on EMA n (%)**			
Received training	18 (75%)	12 (80%)	6 (66.7%)
Did not receive training	1 (4.2%)	0 (0%)	1 (11.1%)
Not stated	5 (20.8%)	3 (20%)	2 (22.2%)
**Personalized Feedback n (%)**			
Received feedback	6 (25%)	1 (6.7%)	5 (55.6%)
Did not receive feedback	5 (20.8%)	3 (20%)	2 (22.2%)
Not stated	13 (54.2%)	11 (73.3%)	2 (22.2%)
**EMA sampling methods**^4^ **n (%)**			
Time Contingent	17 (73.9%)	11 (73.3%)	6 (66.7%)
Signal Contingent	15 (65.2%)	10 (66.7%)	5 (55.6%)
Event Contingent	7 (30.4%)	5 (33.3%)	2 (22.2%)
Passive Monitoring	3 (13%)	2 (13.3%)	1 (11.1%)
**Adherence (% completion)**			
Mean (SD)	69% (17.6)	64% (20.3)	76.7% (7.2)
Median (min-max)	73.9% [30.4–90]	70% [30–90]	76.8% [64–89]
**Renumeration**^3^ **($USD)**			
Mean (SD)	$124 (115)	$150 (121)	$82.2 (99.8)
Median [min-max]	$100 [0–420]	$120 [22–420]	$60 [0–300]
Statistical Analyses - All Studies	n = 43	n = 27	n = 16
**Statistical Model**^4^ **n (%)**			
Mixed effect/Multilevel models	16 (37.2%)	10 (37%)	6 (37.5%)
Structural equation modelling	4 (9.3%)	3 (11.1%)	1 (6.3%)
Time varying equational modelling	4 (9.3%)	3 (11.1%)	1 (6.3%)
Logistic regression	8 (18.6%)	5 (18.5%)	3 (18.8%)
Poisson regression	3 (7%)	1 (3.7%)	2 (12.5%)
Linear regressions/GLMM/GEE	6 (14%)	4 (14.8%)	2 (12.5%)
Machine Learning	5 (11.6%)	4 (14.8%)	1 (6.3%)
Other (ANOVA, Mann-Whitney, t-tests)	7 (16.3%)	4 (14.8%)	3 (18.8%)
**Level of aggregation**^4^ **n (%)**			
Within-person	29 (67.4%)	21 (77.8%)	8 (50%)
Between-person	13 (30.2%)	5 (18.5%)	8 (50%)
Aggregated	11 (25.6%)	7 (25.9%)	4 (25%)
**Missing data strategy n (%)**			
FIML	4 (9.3%)	3 (11.1%)	1 (6.3%)
Multiple imputation	6 (14%)	2 (7.4%)	4 (25%)
None - Complete case	20 (46.5%)	16 (59.3%)	4 (25%)
Not stated	13 (30.2%)	6 (22.2%)	7 (43.8%)

* Other races included: American Indian/Alaska Native, Non-Hispanic/Latino, or Asian or Pacific Islander

1 If race category not reported in study, imputed as 0 for calculations.

2 minimum target assessment period (minimum study duration as per study protocol);

3 Most studies reported max $USD renumeration, with one study converted from AUD to USD according to the exchange rate in year of data collection, one study reporting the minimum guaranteed reimbursement, and three not reporting reimbursements;

4 Sub-categories are not mutually exclusive

**Table 2:** Description of Observational Studies. Separated by clinical location and listed from full-scale to pilot studies, within connected studies. Numbers are consiste across Harvest Plots and Microassays throughout the manuscript and supplementary materials.

#	Author (year)	Setting	Inclusion Criteria	Sample Analyzed (n)	Momentary Antecedent(s) + = positive association − = negative association ∅ = null association	Behaviour(s)	Momentary Consequence(s)	Moderators
**EMERGENCY DEPARTMENT**								
1	Bae et al. (2017)	Emergency Department * *Note ML prediction model (detecting drinking)*	Ages 21–28 reporting hazardous alcohol use (AUDIT-C > = 3 women, >=4 men) and 1 + heavy episodic drinking day in the past month, not seeking treatment for alcohol use	30	56 passive device features extracted, top 20 predictive features listed below (96.6% accuracy) **Environmental Context** Time of Day Day of the Week **Social** Duration of outgoing calls # of correspondents # outgoing calls # missed calls #incoming calls **Movement** # of activities # changes in activities Duration of screen interaction # screen unlocks Battery charge Rotation Accelerometer Radius of Gyration Travel distance Time between keypress # happy emoticons used # of deletions # of insertions	Drinking vs. non-drinking episodes	None	None
2	Bae et al. (2018)	Emergency Department * *Note ML prediction model*	Ages 21–28 reporting hazardous alcohol use (AUDIT-C > = 3 women, >= 4 men) and 1 + heavy episodic drinking day in the past month	30	56 passive device features extracted, top 20 predictive features listed below (98.5% accuracy for non-drinking, 70.2% accuracy for low-risk drinking, 84.4% accuracy for high-risk drinking) **Environmental Context** Weekend vs. Weekday Time of day **Social** Duration outgoing calls # Phone correspondents # Outgoing calls # Missed calls # Incoming calls **Movement Factors** # Activities # Changes in activities Screen duration interaction # Screen unlocks Battery length of charge Max. Magnitude rotation Radius of gyration Travel distance meters Time between keypresses # Deletions # Insertions # Happy emoticons	High-Risk Drinking vs. Low-Risk/Non-Drinking Events	None	None
3	Bae et al. (2023)	Emergency Department * *Note ML prediction model*	Ages 21–25 reporting hazardous alcohol use (AUDIT-C > = 3 < 10 women, >=4 < 10 men) and 1 + heavy episodic drinking day in the past month, not seeking treatment for alcohol use	75	70 passive device features extracted, top 20 predictive features for weekday and weekend models listed below (94.3% weekday accuracy; 95% weekend accuracy) **Environmental Context** Time of day (wkday/wkend) # locations (wkday/wkend) **Movement** Weekday: Latitude (min, max, med, avg, std) Longitude (min, max, med, std) Acceleration magnitude (min, max, mean, med) Weighted stationary latitude Weighted stationary longitude Radius of gyration Total distance Moving time Weekend: Latitude (min, max, med, avg, std) Longitude (min, max, med, avg, std) Acceleration magnitude (min, max, med, mean) Radius of gyration Speed (mean) Total distance #rows of battery	Heavy Drinking Episode	None	Interactions between Time o Day and Radius of Gyration Interactions between Location Coordinates and Day of Week
4	Suffoletto et al. (2017)	Emergency Department	Young adults (21–26) reporting hazardous drinking (AUDIT-C score > = 3 women/ >=4 men) not seeking SUD treatment	10	None	Blood Alcohol Concentration/ Perceived Intoxication	**Cognition** Psychomotor task performance (1) digit symbol substitution task (DSST) −/∅ (2) visuospatial working memory task (VSWMT) ∅/∅	None
5	Suffoletto et al. (2018b)	Emergency Department * *Note ML prediction model*	Aged 21–26, not in emergency for substance use reporting recent hazardous alcohol use (AUDIT-C > = 3 women, >=4 men), not seeking treatment for substance use	10	**Movement Factors** (24) Gait-related features based on accelerometer, gyroscope and magnetometer sensors	Alcohol Use (BAC)	None	None
6	Buu et al. (2020)	Emergency Department	Ages 18–29 with current alcohol users (2–4 times per month in past 6 months) and marijuana users (1 per week in past 6 months)	109 (alcohol) 153 (cannabis)	**Compliance** Measurement Reactivity (Daily vs. Weekly) +/−/∅/∅ Measurement Reactivity (Interactive Voice Response vs. SMS) ∅/∅/−/+ **Environmental Context** Weekend (vs. Weekday) +/+/∅/∅	Alcohol Use / Alcohol Quantity/Cannabis Use / Cannabis Quantity	None	None
7	Suffoletto et al. (2020)	Emergency Department	Aged 18–25 reporting hazardous alcohol use (AUDIT-C > = 3 women, >=4 men) and 1 + heavy episodic drinking day in the past month, not seeking treatment for alcohol use	296	**Attention & Impulsivity** Attention & approach biases ∅	Heavy Episodic Drinking	None	No prior 24-hour drug/alcohol use ∅
MEDICAL CLINIC								
8	Shrier et al. (2012)	Outpatient Medical	Aged 15–24 reporting using cannabis use 2 + times a week	41	**Substance Use** Perceived Availability (fairly easy vs. very easy) +/− Perceived availability impossible/difficult vs. Very easy) ∅/∅ **Affect & Cognition** Positive Affect ∅/+ Negative Affect ∅/+ **Environmental Context** Home (vs school/work) +/∅ Friend’s house (vs school/work) +/∅ Other place (vs school/work) ∅/∅ Midday (vs morning) ∅/+ Evening (vs morning) +/+ Night (vs morning) ∅/+ Weekend (vs weekday) ∅/∅ **Social** Alone (vs. Friends) −/− Parents (vs. Friends) −/∅ Family or other ∅/−	Cannabis Cravings Any/Strength	None	None
9	Shrier et al. (2014)	Outpatient Medical	Aged 15–24 reporting cannabis use 2 + times a week	40	**Affect & Cognition** Positive Affect ∅ Negative Affect +	Cannabis Use Events	None	**Substance Use** Perceived Availability ∅ **Social** Being with Friends ∅
10	Shrier et al. (2013) ^1^	Outpatient Medical	Aged 15–24 reporting cannabis use 2 + times a week	40	**Substance Use** Blunt (vs. Pipe) +/+/∅ Bong (vs. Pipe) +/ ∅/∅ Edible/Vape/Other (vs. Pipe) ∅/∅/∅ **Affect & Cognition** Motives (enhancement, expansion, social vs. cope/conform) ∅/∅/∅ **Environmental Context** Home (vs. Friends) ∅/∅/∅ School/work/other (vs. friends) ∅/∅/∅ Weekends (vs. Weekdays) +/∅/∅ Morning (vs Night) ∅/∅/∅ Midday (vs Night) NR ∅/∅/∅ Evening (vs. Night) +/∅/∅ **Social** Alone (vs. friends) ∅/∅/− Partner (vs. friends) ∅/∅/∅ Parents (vs. friends) ∅/−/−	Cannabis Use Event^1^ Dose / Highest High / Duration	None	None
11	Shrier and Scherer (2014)	Outpatient Medical	Ages 15–24 with 2 + cannabis use events in the past week	36	**Substance Use** Blunt (vs pipe) ∅ Bong (vs pipe) ∅ Other method (vs pipe) ∅ Dose (6 + hits vs 1–5) ∅ Perceived High (above vs. below median) ∅ **Affect & Cognition** Pleasure motives + Coping motives ∅ Social motives + Expansion motives + Conforming motives NR Negative motive (vs. Positive) ∅ External motives (vs. Internal) + **Environmental Context** Friend’s house (vs. home) ∅ Other place (vs. home) ∅ Weekend (vs. weekday) ∅ Afternoon (vs. morning) ∅ Evening (vs. morning) ∅ Early morning (vs morning) ∅ **Social** Alone (vs. other people) ∅ Friends (vs. other people) ∅	Change in reason for cannabis use	None	None
12	Ross et al. (2018)	Outpatient Medical	Aged 15–24, used cannabis 2 + times per week	41	**Environmental Context** Time of Day ∅^D^ Day of Week ∅^D^ **Social** Social (in-person) NR	Cannabis Use	**Affect and Cognition** Positive Affect relative to antecedent ∅ Positive Affect relative to background ∅ Negative Affect relative to antecedent + Negative Affect relative to background +	**Substance Use** Dependence +/+/∅/+ No Dependence 0/−/+/+ **Affect & Cognition** Motives for Use (using to cope/conform vs other) ∅/+/+/+
13	Black et al. (2014)	Outpatient Medical	Aged 15–24, reporting cannabis use 2 + times per week	14	**Substance Use** Methods of Cannabis Use NR **Affect & Cognition** Motives for Use NR Cravings NR Affect NR **Environmental Context** Location NR Time NR **Social** Company (in-person) NR	Cannabis Use	None	None
14	Mennis et al. (2016)	Outpatient Medical	Ages 13–14. No substance use inclusion/exclusion criteria.	139	**Environmental Context** Neighbourhood disadvantage +	Substance use	None	None
15	Mason et al. (2016)	Outpatient Medical	Ages 13–14. No substance use inclusion/exclusion criteria.	248	**Environmental Context** Less risky activity spaces (based on educational attainment, residential stability, housing infrastructure poverty, race, employment) −	Substance Use	None	None
16	Kauer et al. (2009)	Outpatient Medical	Ages 16–24; >7 drinks for women and > 11 drinks for men on 2 + occasions in the past 2 weeks	8	**Affect & Cognition** Negative Affect +^D^ Reasons for Use: get drunk, to relax, or other (vs. feel good, distract) +^D^ **Environmental Context** Time of Day NR Activities NR Location NR **Social** With partner +^D^	Alcohol Use	None	None
MENTAL HEALTH								
17	Stevenson et al. (2022)	Mental Health (partial Hospitalization)	Sample 2: Aged 18–26 attending partial hospitalization psychiatric program using alcohol 2 + times per week in the past month and drinking to cope with moderate-severe anxiety symptoms and high risk for depression	19	**Affect & Cognition** Excited + Sad ∅ Irritable ∅ Stressed ∅ Relaxed ∅ Happy ∅ *Personal Causal Models* Positive Mood Pathway + (subsample) Negative Mood Pathway + (subsample)	Alcohol Use	None	None
18	Selby et al. (2014)	Mental Health (outpatient)	Thoughts of non-suicidal self-injury in the past 2 weeks	30	**Substance Use** Alcohol Use + Drug Use ∅ (+ other non-substance related antecedents)	Self-Injury* (not included in figures/other tables)	None	None
19	Brick et al. (2023)	Mental Health (Inpatient)	Ages 13–18, hospitalized for suicidality (excluding those with psychosis)	62	**Substance Use** Other drug use + **Affect & Cognition** Thoughts of self-harm ∅	Cannabis Use	**Affect and Cognition** Positive Affect + Anger/irritability – Negative Affect ∅	Psychiatric diagnoses (GAD or PTSD) +/∅/∅ (MDD or CUD) ∅/∅/∅
20	Bhushan et al. (2013)	Mental Health (Outpatient)	Aged 15–22 with clinically elevated symptoms of depression and use of cannabis or alcohol	38	**Affect & Cognition** Positive Affect ∅/∅/∅ (- girls) Negative Affect +/ ∅/− Range of Positive Affect −/∅/− Range of Negative Affect −/−/−	Any/Alcohol / Cannabis Use	None	Heavy baseline use status +
21	Weiss et al. (2022)	Mental Health (outpatient focused on psychosis)	Ages 15–25 with high-risk symptoms of or meeting criteria for a psychotic disorder. Excluded those with moderate to severe SUD or substance-induced psychosis.	33	**Substance Use** Recent use substance use NR **Affect and Cognition** Negative Affect − Psychotic Symptoms ∅	Substance Use (nicotine, cannabis, depressants, stimulants)	**Affect and Cognition** Negative Affect + Psychotic Symptoms ∅	None
SUBSTANCE USE								
22	Cleveland and Harris (2010)	Substance Use (Collegiate Recovery Centre / Outpatient)	Members of the collegiate recovery centre at the local university	55	**Affect & Cognition** Negative Affect+ **Social** Hostility+ Insensitivity+ Interference+ Ridicule+	Cravings	None	Avoidance Coping*Negative Triggers + Problem Solving*Negative Affect −
23	Zheng et al. (2015)	Substance Use (Collegiate Recovery Centre/Outpatient)	Members of the collegiate recovery centre at the local university	Total = 39 Cluster 1 = 22 Cluster 2 = 17	**Affect & Cognition** Negative Affect + (both clusters) **Social** Positive Social Experiences (prior day) + (both clusters) Positive Social Experiences (same day) + (both clusters)	Cravings	**Affect and Cognition** Negative Affect ∅ (Cluster 1) + (Cluster 2) Craving + (Cluster 1) + (Cluster 2)	
24	Chiang et al. (2023)	Substance Use (Collegiate Recovery Centre/Outpatient)	Ages 18–29 in recovery from SUD; members of the collegiate recovery centre at the local university	50	**Affect & Cognition** Anger + Fear + Sadness + Guilt ∅	Alcohol and Drug Cravings	None	
25	Zheng et al. (2013)	Substance Use (Collegiate Recovery Centre/Outpatient)	Members of the collegiate recovery centre at the local university who reported smoking and variability in cravings	Pooled = 30 Cluster 1 = 5 Cluster 2 = 25	**Substance Use** Prior day smoking + (Pooled) ∅ (Cluster 1) + (Cluster 2) / ∅ (Pooled) ∅ (Cluster 1) − (Cluster 2) **Affect & Cognition** Cravings ∅ (Pooled) + (Cluster 1) ∅ (Cluster 2) / + (Pooled) + (Cluster 1) ∅ (Cluster 2) Negative Affect ∅ (Pooled) ∅ (Cluster 1) − (Cluster 2) / + (Pooled) + (Cluster 1) ∅ (Cluster 2)	Tobacco Use / Cravings	**Affect and Cognition** Negative Affect ∅ (Pooled) − (Cluster 1) ∅ (Cluster 2) / + (Pooled) ∅ (Cluster 1) + (Cluster 2)	None
26	Comulada et al. (2015)	Substance Use (outpatient)	Ages 13–18 enrolled in SUD treatment	28	**Substance Use** Other Drug Use NR **Affect & Cognition** Negative Affect NR **Environmental Context** Time of Day NR Location NR Activities NR **Social** Social (in-person) NR	Alcohol and Drug Use	Compliance −	None
27	Comulada et al. (2016)	Outpatient Substance Use	Ages 12–18 enrolled in treatment for at least 1 month	28	*variables compared during use to non-use times **Affect & Cognition** Cravings +(daily)/∅(momentary) Positive Affect ∅^D^ Negative Affect ∅^D^ Use Motives +^D^ (wanting to get buzzed for alcohol, wanting to relax for cannabis) **Environmental Context** Hanging out ∅ Weekends (alcohol; vs. weekdays) +^D^ Night (alcohol) +^D^ Night (cannabis and other drugs) ∅^D^ At friend’s house + **Social** With close friends + With people who use substances+	Any substance use (descriptively split by alcohol, cannabis, other drug use	None	None

1 Shrier also reported descriptive comparisons of antecedents for any cannabis use event. Given there were three statistical models, descriptive results not reported in this table.

**Table 3:** Summary of EMIs from intervention studies by clinical setting and primary substance

References of studies and sample size	Sample Descriptions (setting, age group, inclusion)	Research question; Target behaviour, Follow-Up Time	EMI name	Description of EMI	Summary of intervention results	Other notable results
**Emergency Department (alcohol-focused)**						
**MATCH app (k = 4)** #28 Suffoletto and Chung (2025) n = 938 #29 Suffoletto and Chung (2023a) n = 821 #30 Suffoletto and Chung (2023b) n = 297 #31 Chung et al. (2025) n = 344	Emergency Department Emerging Adults Inclusion criteria: Aged 18–25, AUDIT-C score > = 3 for women and > = 4 for men, at least one binge drinking event in past month	Do the different EMIs reduce binge drinking and drinks per drinking day? (directly or indirectly through reduced desire to get drunk or mediated by goal commitment and confidence) Alcohol (drinking and binge drinking events) measured at 14-week follow-up time	PLAN	Youth received feedback on drinking plans and desire to get drunk for 14 weeks. Text messages were sent around the 2 days a week youth were most likely to drink (based on self-reported data at baseline) or could be self-initiated if youth planned to drink that day. They received a reinforcing message if they reported no plan to drink, positive reinforcement if they planned to drink with no desire to get drunk, and a message encouraging reflection and challenging expectations if they reported a desire to get drunk.	No within-group significant effects found No between-group significant effects reported (Suffoletto and Chung, 2025)	**Across all participants (regardless of EMI assignment):** Overall, youth reported alcohol consumption on 16.9% of days when they had no plan to drink (Suffoletto and Chung, 2025). The odds of unplanned drinking were higher for youth who were older, Black, had higher AUDIT-C scores, and higher negative urgency scores. Also higher on weekends and in the presence of friends drinking (Suffoletto and Chung, 2025). Controlling for between-person effects, each unit increase in desire to get drunk predicted greater odds of same-day binge drinking and higher number of drinks per drinking days (Suffoletto and Chung, 2023a). Greater than usual desire to get drunk was associated with lower drinking limit goal commitment and confidence, whereas greater than usual goal commitment and confidence were associated with lower likelihood of same-day binge drinking (Suffoletto and Chung, 2023b).
			USE	Youth received feedback on drinking quantity with the goal of shifting perceptions of binge drinking from positive to negative. If participants reported no drinking during the week, they received positive reinforcement. If drinking was below binge level (< 4 standard drinks for women, < 5 standard drinks for men), they received a message framing it as low risk. If they reported binge drinking, they received a message labelling it as such and addressing norms/risks.	USE showed significantly reduced odds of unplanned drinking days compared to TRACK (Suffoletto and Chung, 2025) USE showed significant mediation through desire to get drunk compared to TRACK; 35.9% of USE’s effect on reduced binge drinking was explained by lowering desire to get drunk (Suffoletto and Chung, 2023a).	
			GOAL	Youth were prompted to set personal drinking goals, tips to achieve goals, and feedback on goal attainment If no goal was set, they received a message to reduce resistance. If a goal was set, they received encouragement and a protective strategy tip. Responses about confidence levels triggered messages to boost self-efficacy. The next day, youth received feedback to support success or reframe failure.	GOAL reduced negative alcohol consequences compared to TRACK (Suffoletto and Chung, 2025) GOAL had greater reduction in binge drinking days for females who perceived greater risk associated with binge drinking and greater number of friends drinking to intoxication compared to females in COMBO (Chung et al., 2025). White males in GOAL had greater reduction in binge drinking compared to white males in COMBO (Chung et al., 2025).	
			COMBO	Youth received a combination of all above interventions – they received all SMS and feedback interventions/options.	COMBO showed significantly reduced odds of unplanned drinking days and negative alcohol consequences compared to TRACK (Suffoletto and Chung, 2025) COMBO showed significant mediation through desire to get drunk compared to TRACK; 34.4% of COMBO’s effect on reduced binge drinking was explained by lowering desire to get drunk (Suffoletto and Chung, 2023a). 60.8% of COMBO’s effect on reducing drinks per drinking day were explained by lowering desire to get drunk (Suffoletto and Chung, 2023a). Females in COMBO who perceived lower risk of binge drinking-related harm and no cannabis use in past 3 months had greater reductions in drinking outcomes compared to their counterparts in GOAL (Chung et al., 2025). Non-white females in COMBO who reported lower risk of binge drinking-related harm and any cannabis use in past 3 months had greater reductions in drinking outcomes compared to their counterparts in GOAL (Chung et al., 2025) Non-white males had greater reductions in drinking outcomes compared to their counterparts in GOAL (Chung et al., 2025).	
			TRACK (control)	Youth were prompted to self-monitor drinking plans, desire to get drunk, and drinking without any feedback or reflection prompts.	This was the control group across all analyses.	
**Texting to Reduce Alcohol Consumption (TRAC) Trial** #32 Suffoletto et al. (2014) n = 765 #33 Suffoletto and Chung (2016) n = 384	Emergency Department Emerging Adults Inclusion criteria: Aged 18–25, AUDIT-C score > = 3 for women and > = 4 for men	Does the TRAC Trial encourage lower drinking consumption among young adults? Alcohol (drinking and binge drinking events measured at 3-month follow-up time	SMS Assessments + Feedback (SA+ F)	Over the duration of 12 weeks, youth were asked on Thursday about their drinking plans for the upcoming weekend and received real-time feedback via SMS. On Sunday, youth were asked to report the most drinks they had on the weekend. Depending on responses, youth were provided feedback via SMS. On both Thursday and Sunday, feedback depended on drinking plans and focused on either strengthening low-risk drinking plans or promoting reflection on their plan if not considered a low-risk goal.	Within-group decreases were noted in the number of binge drinking days and number of drinks per drinking day from baseline to 3 months (Suffoletto et al., 2014) Those in the SA + F group had fewer youth reporting any binge drinking in last 30 days from baseline to 3 months compared to SA and control groups (Suffoletto et al., 2014) SA+ F participants were more likely to not report any binge drinking at 3-months compared to control participants (Suffoletto et al., 2014) SA + F participants reported less drinks consumed per weekend over the 12 weeks compared to the SA group (Suffoletto et al., 2014)	**Across all participants (regardless of EMI assignment):** From latent class analysis: The *“not willing to limit drinks*” class exhibited the least reductions in alcohol consumption and was more likely to include White individuals and those with higher baseline drinking severity. The *“planned not to drink”* class had the greatest reductions in alcohol consumption (Suffoletto and Chung, 2016).
			SMS Assessments (SA)	Youth responded to questions about drinking each Sunday, but did not receive any feedback	Increases in number of binge drinking days and number of drinks per drinking day from baseline to 3 months (Suffoletto et al., 2014)	
			Control	Youth did not receive any text messages.	Increases in number of binge drinking days and number of drinks per drinking day from baseline to 3 months (Suffoletto et al., 2014)	
**Texting to Reduce Alcohol Consumption 2 (TRAC2) Trial** #34 Suffoletto et al. (2018a) n = 38	Emergency Department Emerging Adults Inclusion criteria: aged 18–25, AUDIT-C score > = 3 for women and > = 4 for men	Do subjects who opt in to the TRAC2 Trial for longer periods have greater reductions in alcohol related outcomes? Alcohol (drinking and binge drinking events measured at 3-month Follow-up time	Single arm intervention	Youth were asked about their weekend drinking plans and willingness to commit to a drinking limit goal. Instead of the youth setting their own goal, an algorithm was used to set a personalized drinking limit based on the average number of drinks the youth consumed over the previous two weekends. If the average exceeded 10 drinks, the goal was capped at 10. If the average was between 5 and 10, the limit was set to one less than the reported average.	Participants were categorized into groups based on the duration of their voluntary participation in the intervention (4, 8, 12, or more than 12 weeks). The 4-week, 8-week, and 12-week groups significantly reduced their drinking over their designated EMA periods Only the 12-week group demonstrated a significant difference from baseline to 3-month follow-up in median number of negative consequences	**Across all participants (regardless of EMI assignment):** Slowly decreasing participants’ drinking limits over time appeared to be more palatable and successful relative to larger reductions in typical drinking amounts. Although earlier weeks prompted some participants to set a goal higher than the binge threshold, safety regarding setting drinking goals at levels associated with negative outcomes was a time limited issue. By week 4, no participants were setting goal limits above the binge threshold.
**Outpatient medical clinic (cannabis-focused)**						
**MOMENT (k = 2)** #35 Shrier and Harris (2023) n = 68 #36 Shrier et al. (2018) n = 70	Medical Outpatient Emerging Adults Inclusion Criteria: Aged 15–24, used cannabis 3 times per week	Does momentary mindfulness increase after intervention, compared to control, and after controlling for differences in momentary context? What are the associations of momentary mindfulness with momentary affect and cannabis desire? Cannabis use measured at 3-month follow-up time	MOMENT	MET (motivational enhancement therapy) + EMA with assessments: two in-person MET sessions (1 week apart), with 1 week of EMA/EMI at baseline and 1 week of EMA/EMI at 3-month follow up using mobile self-monitoring with feedback messages Youth completed momentary reports of top 3 triggers for use (from a list of affective and social triggers identified during MET), desire for cannabis, or effort to avoid use. Motivational messages were sent after EMAs.	Mindfulness/attention (MAA) scores increased pre vs post intervention (Shrier and Harris, 2023) Momentary marijuana desire declined more in MOMENT vs. MET-only (Shrier et al., 2018) Marijuana use following a targeted context or behavior was less likely in MOMENT vs. MET-only (Shrier et al., 2018)	**Across all participants (regardless of EMI assignment):** Across arms, participants reported significantly lower marijuana use, desire, and problems at follow-up compared to baseline (Shrier et al., 2018) A higher MAA score was associated with lower momentary negative affect and lower momentary cannabis desire. No significant relationship between MAA and positive affect was found (Shrier and Harris, 2023) Participants highly rated acceptability; their comments reflected changing motivation and behaviour (Shrier et al., 2018)
			MET + No message	MET + EMA without messages: two in-person MET sessions and completed smartphone reports without messages (1 week of EMA at baseline and 1 week of EMA at 3-month follow up)	Marijuana use following a targeted context or behavior was less likely in No-messages vs. MET-only (Shrier et al., 2018)	
			MET-only	two in-person MET sessions, no EMAs sent	Within-person mindfulness/attention scores did not increase pre vs post intervention (Shrier and Harris, 2023)	
**Mental health program (alcohol-focused)**						
**Choosing Healthy Options in Coping with Emotions (Project CHOICE)** #37 Blevins et al. (2021) n = 20 #38 Stevenson et al. (2020) n = 20	Partial hospitalization at a private psychiatric institution Emerging Adults Inclusion criteria: Aged 18–25, past month alcohol use > = 2 times weekly, reported drinking to cope, moderate to severe anxiety symptoms, high risk for depression	What impact does the CHOICE intervention have on drinking motives, mood, coping strategy and alcohol outcomes? What is the momentary relationship between mood and alcohol use? Alcohol, motives measured at 6-week follow-up time	Single arm coping intervention	Youth completed a 6-week EMA/EMI after completing 5–10 days partial hospital treatment for anxiety, depression, or other disorders. Orientation session (in-person) with a PhD-level clinical psychologist included normative feedback on youth’s alcohol use and coping motives (compared to peers), education on the risks of using alcohol to cope, and the development of personalized alternative coping strategies. Non-substance related coping strategies included: distract, efforts to fix, emotional support, avoid family, social media, positive reframe, give up, eat food, ask for help, make a plan, exercise, pornography, and sex. The EMA/EMI protocol involved 4 assessments per day, prompted at a random time, or on demand, asking about alcohol use since the last assessment and current mood. If youth reported negative mood or intent to drink, an individualized, self-chosen, coping message (identified from in-person assessment) was sent. Messages were CBT-based and aimed to reduce drinking to cope by reinforcing helpful strategies.	At baseline, drinking and coping motives were highly correlated (Blevins, 2021) From baseline to 6-week follow-up, participants had significant decreases in percent days of alcohol use, percent days of binge drinking, percent days drinking to cope, problematic drinking and coping motives related to anxiety, depression, social and conformity factors (Blevins et al., 2021, Stevenson et al., 2020) Number of coping strategies was negatively related to quantity of alcohol consumed (Stevenson et al., 2020). The interaction between negative mood and coping suggested individuals were less likely to drink in response to negative mood when they used more coping strategies (Stevenson et al., 2020).	**Feasibility:** Results indicated high levels of feasibility and acceptability (Blevins et al., 2021). All participants reported that they would continue to monitor their drinking. Several themes emerged in the qualitative interviews: identifying and tracking emotions, insights about their drinking habits, the role of emotions in their drinking, and the use of coping skills (Blevins et al., 2021).
**Substance use program (cannabis, tobacco, general substance)**						
**ACHESS (Addiction Comprehensive Health Entrance Support System)** #39 Dennis et al. (2015) n = 29	Residential substance- use treatment program Adolescents Inclusion Criteria: discharge from residential treatment, aged 18 or below	Can EMAs and recovery support EMIs via smartphone be used to predict substance use in the subsequent week? General Substance Use measured at 7 days follow-up time	Single arm intervention of a menu of EMIs	EMA questions focused on youths’ experiences in the past 30 minutes, related to emotions, activities, location, social context, cravings, exposure to substances, and ability to resist drug or alcohol use. Optional self-initiated EMIs were available anytime, that offered tools for recovery support, relaxation, motivation, and social networking.	Substance use in the past 30 minutes (vs. not) was the single best predictor of any subsequent use in the next 7 days The next best predictor was 20 combined ratings of internal and external risk/protective factors from the EMA (i.e. people, places, feelings, activities, current use, exposure to substances, pain, withdrawal craving, ability to resist using). Youth using 2 or more EMIs within an hour of the EMA were significantly less likely than those who did not to use drugs or alcohol within the 7 days	**Across all participants (regardless of EMI assignment):** Compared to the recognized risk observations (youth who were not using and recognized risk of future use), substance use in the following seven days was significantly more likely after unrecognized risk observations and current use observations. Youth who recognized their risk for use or were already using were more likely to use substances, compared to youth who weren’t using substance and understood the risks of future use.
**Ecological momentary motivational enhancement therapy (EM-MET)** #40 Darharaj et al. (2025) n = 52 *Note: Iranian adaptation of MOMENT	Specialized substance-use treatment centres Emerging adults Inclusion Criteria: Age 18–24, cannabis use disorder diagnosis, cannabis use 3 times per week	Does the intervention (EM-MET) reduce cannabis use temptation and dependence severity among young adults with Cannabis Use Disorder (CUD)? Is the mobile-based EM-MET feasible and acceptable for young adults with CUD? Cannabis use measured at 1-month and 2-month follow-up times	EM-MET	Four twice-weekly individual 90-minute sessions focused on 1) increasing awareness of the prevalence and severity of cannabis use issues, 2) identifying goals and values, 3) developing awareness of discrepancies between cannabis use and the goals and values, 4) encouraging intrinsic motivation to reduce or abstain from cannabis use, and 5) creating a plan for change During the first MET, youth selected their top four triggers for cannabis use which were used in the EM-MET intervention. The SMS EMA prompted youth four times per day over two weeks. If participants reported one of their triggers, they immediately received a call from a therapist, who delivered MET through the phone to address the situation and trigger.	Youth found the EM-MET to be feasible, acceptable, and useful Compared to MET-alone, the EM-MET showed significantly greater reductions in temptation and dependence severity over time	**Across all participants (regardless of EMI assignment):** Overall decline in desire to use cannabis, however some fluctuation from 2nd to 10th day Clear decline in days of cannabis use and frequency of cannabis use over two weeks of EMA intervention
			MET	The four previously described in-person 90-minute MET sessions only, without SMS-EMA.	Within group decline in temptation and dependence scores	
**ASC (Agenda de sommeil et de comportements) mobile app** #41 Pitrat et al. (2024) n = 36	Substance Use Clinic Adolescents Inclusion Criteria: aged 12–18, attending treatment for cannabis or gaming disorder, access to smartphone	What is the perceived impact using the app to complete behavioural data for young people with addictive behaviours? Cannabis use measured at 15-day follow-up time	App Diary	Youth used an app diary for 15 days to capture real-time frequency and duration of addictive behaviours (gaming and/or cannabis), Assessments also captured time and quality (patient recorded) of sleep. Data was then used in session with clinicians	A median of 67% of information was completed in the intervention. In patients with cannabis addiction, a median of 30% of information was completed. The patient’s knowledge, attitudes, intentions to change, behaviour change and help seeking related to the use of the diaries appeared higher in the app compared to paper group. Participants who completed app diaries regularly provided more information for use during consultations with physicians compared to participants who completed paper diaries.	**Feasibility:** The app had high levels of engagement, functionality, aesthetics and subjective appreciation.
			Paper Diary (control)	Youth used paper diary for 15 days and collected the same data as the app diary, on addictive and sleep behaviours.	A median of 10% of information was completed in the control. In patients with cannabis, a median of 0% of information was completed.	
**SMOKING Intervention** #42 Mason et al. (2015a) n = 200 #43 Mason et al. (2015b) n = 172	Outpatient Substance Use Clinic Adolescents Inclusion criteria: aged 14–18, score above cut-point on modified version of Fagerstrom Tolerance Questionnaire for nicotine dependence	Within a tobacco reduction intervention, to what extent does peer smoking behaviour influence readiness to change? Is craving significantly reduced? Tobacco/Nicotine measured at 5- and 6-month follow-up times. 5 months for readiness to stop smoking and 6 months for friends smoking and current smoking.	Integrated motivational interviewing with social network counseling	Youth received a 5-day integrated MI text message-based intervention with social network counseling. The intervention focused on rapport building, personalized feedback on tobacco use, social network reflection and feedback, and summary and goal setting. The social network aspect helped youth reflect on influence of peers and consider small changes in how and with whom they spend their time. Feedback messages were personalized using data from participants’ baseline assessment and their responses during the intervention. On day 2, youth’s tobacco use is reviewed, and on day 3 youth are provided with age-specific normative feedback.	Reductions in stress on craving post-intervention at 1 week was sustained and became significant between months 2 and 4 (Mason et al., 2015b) The intervention reduced the impact of baseline nicotine dependence on craving, as participants in the intervention group had a steady decline in craving over the 6-month study duration (Mason et al., 2015b)	**Across all participants (regardless of EMI assignment):** Readiness to stop smoking had an indirect and negative effect of treatment on cigarettes smoked for adolescents with fewer friends smoking, but not for those with more friends smoking (Mason et al., 2015a) Baseline dependence and momentary stress predicted momentary craving (Mason et al., 2015b)
			Control	Participants received text messages covering general (diet, exercise and study habits) health habits	During months 2 to 3, the association between stress and craving was significantly stronger among the control group (Mason et al., 2015b)	

Acronyms: CBT (cognitive behavioural therapy); EM – MET (Ecological momentary motivational enhancement therapy); MET (motivational enhancement therapy; MI (motivational interviewing); SA (SMS Assessments); SA + F (SMS Assessment + Feedback)

## Highlights

- EMA/EMIs show promise for youth substance use in clinical settings
- Momentary predictors of use across substances and settings were mixed
- Negative affect, substance use, time/day, and social context were most consistently related
- EMIs incorporating feedback, goal setting, and coping strategies reduced substance use
- Evidence remains exploratory with notable methodological limitations
